# The Effectiveness and Safety of Tibial-Sided Osteotomy for Fibula Untethering in Lateral Close-Wedge High Tibial Osteotomy: A Novel Technique with Video Illustration

**DOI:** 10.3390/medicina61010091

**Published:** 2025-01-08

**Authors:** Keun Young Choi, Man Soo Kim, Yong In

**Affiliations:** Seoul St. Mary’s Hospital, The Catholic University of Korea, Seoul 06591, Republic of Korea; heaxagon@hanmail.net (K.Y.C.); kms3779@naver.com (M.S.K.)

**Keywords:** lateral close-wedge high tibial osteotomy, protective effect, popliteus muscle, popliteal neurovascular structure, peroneal nerve, recurrent branch of anterior tibial artery

## Abstract

*Background and Objectives*: Despite its advantages, lateral close-wedge high tibial osteotomy (LCWHTO) requires proximal tibiofibular joint detachment (PTFJD) or fibular shaft osteotomy for gap closing. These fibula untethering procedures are technically demanding and not free from the risk of neurovascular injuries. Our novel fibula untethering technique, tibial-sided osteotomy (TSO) near the proximal tibiofibular joint (PTFJ), aims to reduce technical demands and the risk of injury to the peroneal nerve and popliteal neurovascular structures. The purposes of this study were to introduce the TSO technique and compare the complexity and safety of TSO with those of radiographic virtual PTFJD, which is defined based on radiographic landmarks representing the traditional PTFJD technique. *Materials and Methods*: Between March and December 2023, 13 patients who underwent LCWHTO with TSO for fibula untethering were enrolled. All patients underwent MRI preoperatively and CT scanning postoperatively. The location of the TSO site on the postoperative CT scans was matched to preoperative MRI to measure the shortest distance to the peroneal nerve and popliteal artery. These values were compared with estimates of the distance between the PTFJ and neurovascular structures in the radiographic virtual PTFJD group. The protective effect of the popliteus muscle was evaluated by extending the osteotomy direction toward the posterior compartment of the knee. *Results*: The TSO procedure was straightforward and reproducible without producing incomplete gap closure during LCWHTO. On axial images, the distances between the surgical plane and the peroneal nerve or popliteal artery were significantly longer in the TSO group than in the radiographic virtual PTFJD group (both *p* = 0.001). On coronal and axial MRI, the popliteus muscle covered the posterior osteotomy plane in all patients undergoing TSO but did not cover the PTFJD plane in the radiographic virtual PTFJD group. *Conclusions*: Our novel TSO technique for fibula untethering during LCWHTO is reproducible and reduces the risk of neurovascular injury by placing the separation site more medially than in the PTFJD procedure.

## 1. Introduction

Valgus-producing high tibial osteotomy (HTO) is a well-accepted treatment modality in active patients with varus malalignment and symptomatic medial unicompartmental osteoarthritis (OA) of the knee [1]. Traditionally, lateral close-wedge HTO (LCWHTO), introduced by Coventry et al. [2], has been considered the gold standard osteotomy procedure for patients with varus knee alignment. However, medial open-wedge HTO (MOWHTO) has recently surpassed LCWHTO in terms of the number of procedures performed [3]: MOWHTO is easier to perform and tends to more reproducibly acquire an adjustable alignment change [4]. While LCWHTO has certain advantages when compared to MOWHTO, including greater initial postoperative stability, a faster return to weight bearing, favorable bone union, greater ability to address large correction with a lower risk of nonunion, less leg-length discrepancy (LLD), and lower risk of patellofemoral OA and increased tibial slope [5], the LCWHTO procedure can be technically demanding and may not allow precise adjustment [6]. Furthermore, LCWHTO requires an additional fibula untethering procedure that poses a risk of injury to the common peroneal nerve and/or popliteal neurovascular structures that must be considered when selecting a treatment option for varus malaligned medial OA, especially for novice surgeons [7].

Three surgical options to manage the fibula in LCWHTO have been described: proximal tibiofibular joint detachment (PTFJD), fibular head resection, and fibular shaft osteotomy [2,3,8,9]. Although the surgeon generally selects the preferred option, PTFJD has been reported to have the debatable risk of lateral laxity [8,10,11], iatrogenic fibular head fracture resulting in early postoperative proximal tibiofibular joint (PTFJ) arthritis [3]. In addition, fibular head resection requires an additional procedure to reattach the biceps femoris tendon and lateral collateral ligament to the fibular neck and may pose an increased risk of injury to the common peroneal nerve [3,9]. Furthermore, fibular shaft osteotomy was also reportedly associated with a greater risk of nonunion of the fibula shaft, peroneal nerve palsy, and pain at the osteotomy site [5,8,9,12].

We devised a novel tibial-sided osteotomy (TSO) technique for fibula untethering during LCWHTO, (Video S1) that is technically straightforward and reduces the risk of injury to the peroneal nerve and/or popliteal neurovascular structures. Thus, the purposes of this study were to introduce our novel TSO technique and compare the safety of the TSO and radiographic virtual PTFJD techniques by measuring the distances from the surgical site to the neurovascular structures using preoperative MRI and postoperative CT scans.

## 2. Materials and Methods

### 2.1. Patients

Between March and December 2023, we prospectively enrolled 13 consecutive patients 70 years of age or younger with isolated medial compartment OA of the knee who were scheduled to undergo an LCWHTO procedure performed by a single surgeon. All radiographic evaluations were conducted retrospectively. The HTO procedure was contraindicated if a patient had symptomatic OA in the lateral compartment or the patellofemoral joint of the knee, inflammatory arthritis, a flexion contracture of 15° or more, knee range of motion less than 120°, joint instability, or a history of knee joint infection [13]. At our institution, the decision to perform MOWHTO or LCWHTO was mainly based on the amount of correction required, which was calculated from preoperative standing radiographs of the lower extremities. Usually, an LCWHTO was performed in patients with a correction angle of 13° or more, an excessive tibial slope, or LLD, or for patients in which there was a risk of aggravation of patellofemoral OA. During the study period, 18 MOWHTOs were performed. The average preoperative hip–knee–ankle (HKA) axis was 11.0. The mean medial proximal tibia angle (MPTA) and mechanical lateral distal femur angle (mLDFA) were 80.7 and 87.6, respectively. The mean correction angle of the patients who underwent MOWHTO was 9.4° (range 6−12°), and that of the patients who underwent LCWHTO was 14.8° (9−18.5°). A patient with a correction angle of 9° underwent LCWHTO because of an acute skin wound on the medial side of the proximal tibia. The mean age of the study cohort was 57.8 years (54−63 years), and there were 2 males and 11 females. These 13 patients underwent MRI evaluation preoperatively to identify intraarticular pathologies, including meniscal tears and cartilage defects. The study design was approved by the institutional review board of our hospital (MC23EADI0064). All patients were informed about the requirements of the study and provided informed consent. This study was supported by the Research Fund of Seoul St. Mary’s Hospital, the Catholic University of Korea.

### 2.2. Surgical Technique

The patient was placed in the supine position on the operating table with an appropriate tourniquet applied over the cast padding. The surgical procedure was performed under general anesthesia with the tourniquet inflated to 300 mmHg. In our institution, LCWHTO was routinely performed under general anesthesia. And a torniquet was also routinely used with 300 mmHg pressure to prevent unintended bleeding. Routine arthroscopic examination was carried out using the anteromedial, anterolateral, and superomedial portals. An arthroscopic examination of all three compartments was performed, and the status of the menisci was determined using a probe.

**Figure 1:**
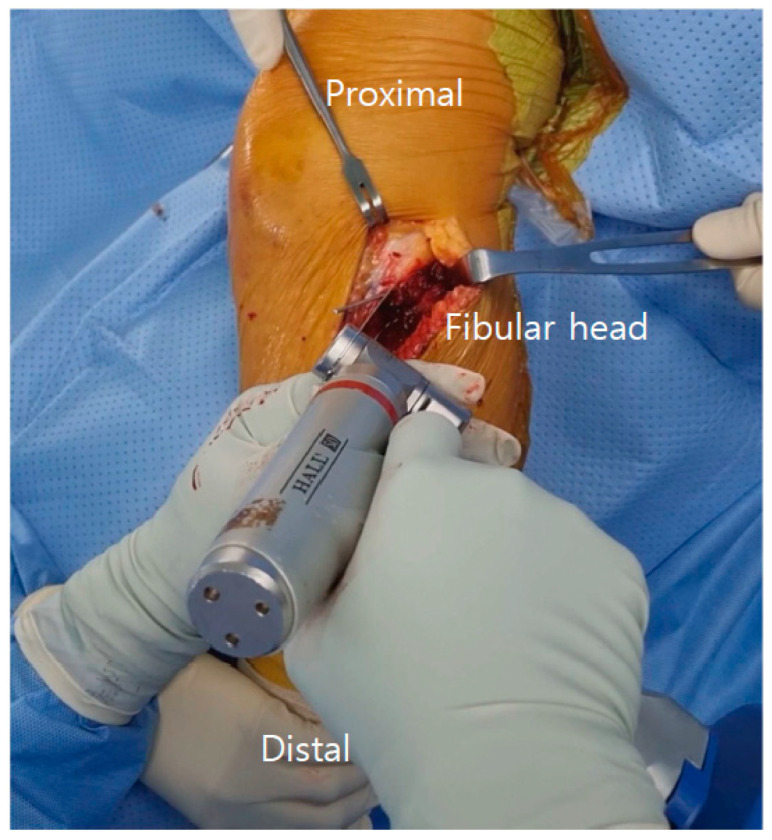
The left knee is shown. A guiding Kirschner wire was inserted 5 mm medial to the PTFJ to secure the articular cartilage and anterior PTFL. Using a sagittal saw, a guiding fissure was made from the near cortex of the proximal tibia to the cancellous bone. PTFJ: proximal tibiofibular joint; PTFL: proximal tibiofibular ligament.

**Figure 2:**
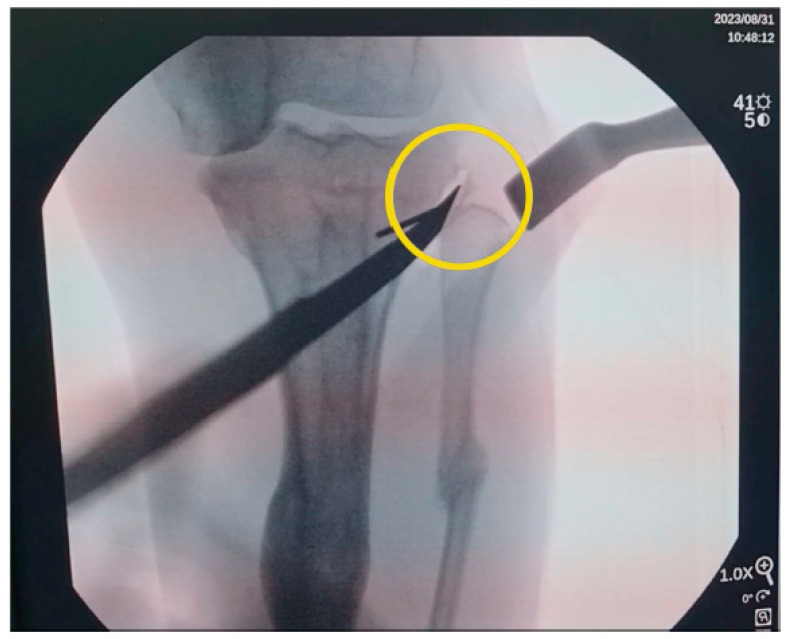
Under fluoroscopic imaging, a small freely movable detached bone fragment was identified at the tibial side (yellow circle).

**Figure 3:**
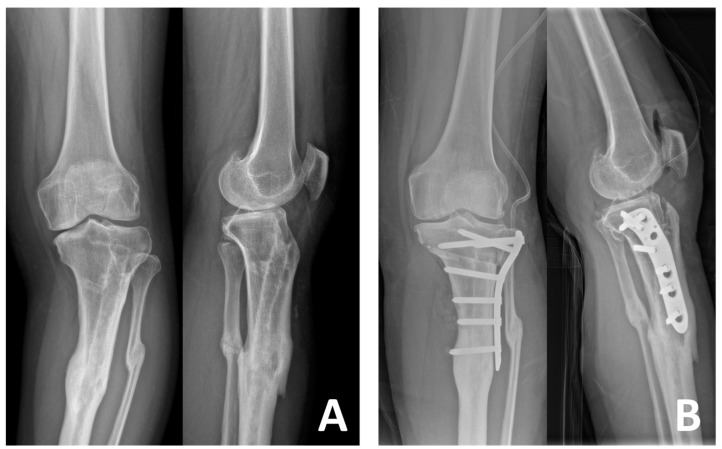
Preoperative (**A**) and postoperative (**B**) radiographs of a 42-year-old female patient who underwent LCWHTO using the TSO technique. LCWHTO: lateral close-wedge high tibial osteotomy; TSO: tibial-sided osteotomy.

For biplanar LCWHTO, a curvilinear incision was made between Gerdy’s tubercle and the fibular head and extended distally following the anterior crest of the tibia, and the tibialis anterior muscle was dissected subperiosteally. First, the PTFJ and anterior proximal tibiofibular ligament (PTFL) were exposed. Our novel technique for fibula untethering was performed utilizing an additional mini-osteotomy on the tibial side of the PTFJ. For this procedure, a Kirschner wire (K-wire) was inserted under fluoroscopic guidance starting at the tibial cortex at a point 5 mm medial to the PTFJ to secure the PTFJ articular cartilage and anterior PTFL. The K-wire was guided parallel to the joint line on the sagittal plane and about 30° oblique medially on the coronal plane towards the popliteus muscle. By leaving the osteotomy plane covered by the popliteus muscle, this placement was found to preserve the PTFJ and not injure the popliteal vessels. Using a sagittal saw, a guiding fissure was made from the near cortex of the proximal tibia to the cancellous bone (Figure 1). After sawing to the near cortex, TSO of the far cortex was performed using a curved osteotome. By levering the osteotome in a medial-to-lateral direction under the fluoroscope, separation of the tibia and the small tibial-side bone fragment with the fibula head could be confirmed, which indicated complete untethering of the fibula (Video S1 and Figure 2). Patients included in this study were confined to a normal degree LDFA and small MTPA with varus malalignment. So, the Paley’s center of rotation of angulation (CORA) was on the proximal metaphyseal area of the tibia. The LCWHTO is an established surgical procedure accepted as one of the gold standard procedures for correcting medial compartment OA with tibia vara. The starting point was located between proximal and distal holes of a TOMOFIX^TM^ Lateral High Tibia Plate (DePuy Synthes, Zuchwil, Switzerland) to ensure the proper location of the plate. And the ending point was 1 cm below the medial plateau of the proximal tibia to avoid MCL injury. The TOMOFIX^TM^ Lateral High Tibia Plate was placed temporarily, and the level of proximal tibial osteotomy was marked with a surgical marking pen. Two K-wires were placed at the level of the tibial plateau. The location of the distal tibial osteotomy was marked according to preoperative calculations of the size of the bone wedge to remove. Two additional K-wires were inserted heading to the hinge point. Posterior to the tibial tubercle, an oblique coronal osteotomy procedure was performed following the line extending from the patella tendon. The proximal and distal tibial osteotomies were performed using a sagittal saw along each of the two K-wires to preserve the far medial cortex and periosteum. The wedge of bone was removed using a pituitary rongeur, and the far medial cortex was carefully drilled with a K-wire and decorticated using an osteotome. A valgus force was gently applied to the level of the osteotomy until the upper and lower osteotomy surfaces contacted each other. A cable was used for an intraoperative assessment of the alignment. The proximal and distal parts were rigidly fixed with a TOMOFIX^TM^ Lateral High Tibia Plate and screws. Figure 3 shows preoperative and postoperative radiographs.

A quadriceps-setting exercise and continuous passive motion began on the first postoperative day. Partial weight bearing with crutches was allowed 4 weeks after surgery, and full weight bearing was started 6 weeks after surgery. The same protocol was applied to all patients, including those who underwent meniscal or cartilage procedures.

### 2.3. Evaluation

To evaluate the safety and ease of performing our novel TSO for fibula untethering, a radiographic virtual conventional PTFJD group was set as the control group.

The radiographic virtual PTFJD group was defined based on radiographic landmarks. If an ideal genuine PTFJD procedure was performed, the dissociation plane should be located between the anterior PTFJ to the posterior end of the PTFJ. According to this hypothesis, evaluations of the radiographic virtual PTFJD group were conducted while assuming the dissociation plane was located on the extended line from the anterior to posterior PTFJ.

**Figure 4:**
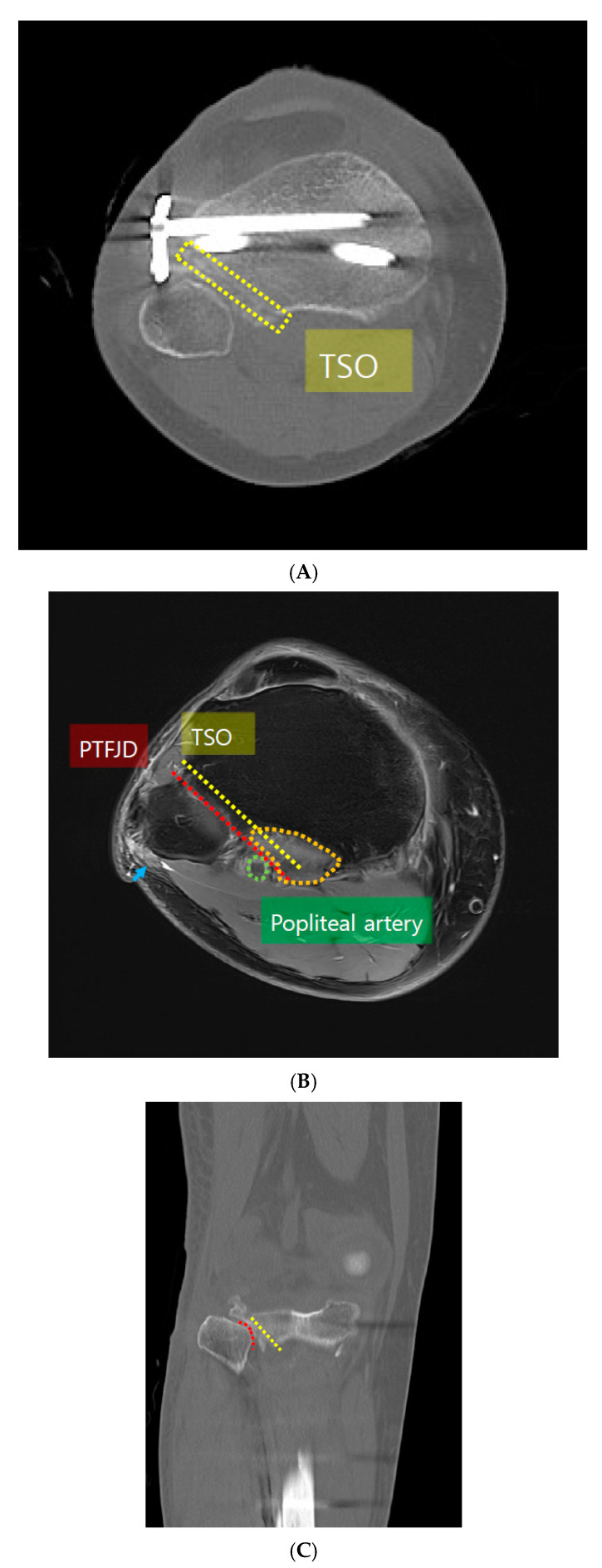

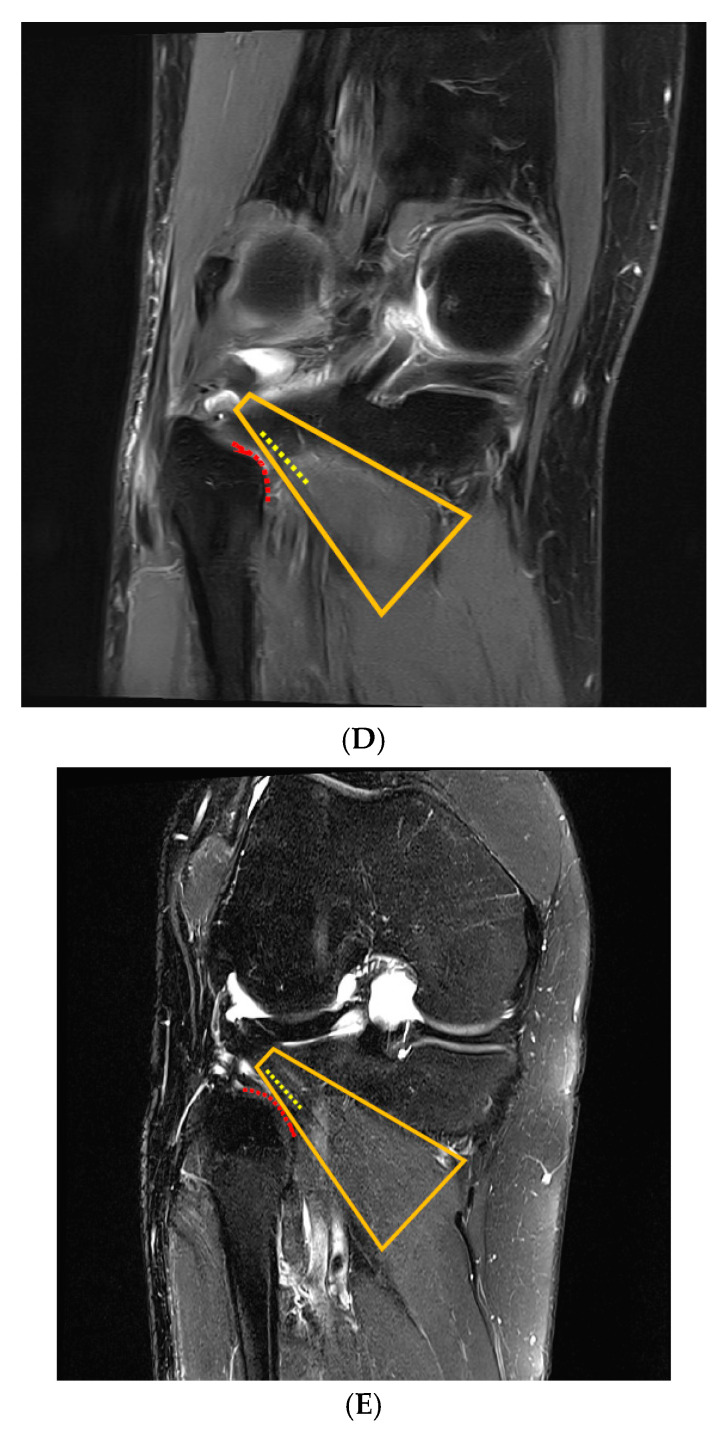
(**A**) An axial image from the postoperative CT scan. On CT scans with the knee in full extension, the osteotomy plane (yellow dotted box) adjacent to the proximal tibiofibular joint is located on the more medial side parallel to the proximal tibiofibular joint. TSO: tibial-sided osteotomy. (**B**) On the axial MRI of the knee in full extension, the TSO plane (yellow dotted line), defined on the corresponding postoperative CT scan, is located more medially than the PTFJD plane (red dotted line) and located farther from the peroneal nerve (blue arrow). A line extending from the TSO plane is covered by the popliteus muscle (orange dotted line), whereas the PTFJD is not, indicating that the popliteus muscle does not protect the popliteal neurovascular structures during PTFJD with the knee positioned in full extension. TSO: tibial-sided osteotomy; PTFJD: proximal tibiofibular joint detachment. (**C**) On the coronal image of the postoperative CT with the knee in full extension, the TSO plane (yellow dotted line) aside the PTFJ (red dotted line) is confirmed. TSO: tibial-side osteotomy; PTFJ: proximal tibiofibular joint. (**D**) On the coronal MRI of the knee in full extension, the TSO plane (yellow dotted line) defined on the corresponding postoperative CT scan is located more medially compared to the PTFJD plane (red dotted line). The TSO plane is covered by the popliteus muscle (orange line), whereas the PTFJD is not, indicating that the popliteus muscle does not protect the popliteal neurovascular structures during PTFJD with the knee positioned in full extension. TSO: tibial-side osteotomy; PTFJD: proximal tibiofibular joint detachment. (**E**) Flex 90 MRI. On the coronal MRI of the knee in 90-degree flexion, the TSO plane (yellow dotted line) defined on the corresponding postoperative CT scan is located more medially than the PTFJD plane (red dotted line). The TSO plane is covered by the popliteus muscle (orange line), whereas the PTFJD is not, indicating that the popliteus muscle does not protect the popliteal neurovascular structures during PTFJD with the knee positioned in 90-degree flexion. TSO: tibial-side osteotomy; PTFJD: proximal tibiofibular joint detachment.

With their consent, postoperative CT scans were performed on all 13 patients to identify the position and status of the osteotomy. The TSO plane was determined on the postoperative CT scan, and the corresponding radiographic virtual plane was identified on the preoperative MRI of the same patient to measure the distance from the surgical plane to the peroneal nerve and popliteal artery that were not definitively seen on the postoperative CT scan (Figure 4A,B). The position of neurovascular structures can be evaluated more accurately using MRI. However, during the MRI scan, even though the metal reduction protocol is adapted, the artifact can disturb authors locating the positions of the neurovascular structures. So, the plane of TSO was evaluated on the postoperative CT scan, and it was embedded to the preoperative MRI. The shortest distance from the extended line of the TSO plane (TSO group) or PTFJ (PTFJD group) to the peroneal nerve and popliteal artery wall were measured on axial images. Although PTFJD was not actually performed in the control group, PTFJD could be assured because the PTFJ could be identified on both the CT scan and MRI (Figure 4B).

The popliteus muscle is known to have a debatable potential to protect the popliteal neurovascular structures during a corrective osteotomy procedure [14]. This protective effect by the popliteus muscle against the osteotome toward the posterior neurovascular structure during the TSO or PTFJD was evaluated on coronal and axial MRI and matched with the postoperative CT scan images (Figure 4A–D).

### 2.4. Image Analyses

Two orthopedic surgeons performed all measurements retrospectively in a step-by-step manner. The final values were the averages of the two evaluations. Each researcher assessed every radiographic variable on two occasions at least two weeks apart. Intra- and inter-observer reliability for each measurement was expressed as an intraclass correlation coefficient (ICC).

First, the two orthopedic surgeons assessed the postoperative CT images of the knee, which were used as reference standards for the TSO or radiographic virtual PTFJD plane. The reviewers evaluated the three-dimensional direction and depth of the TSO plane or radiographic virtual PTFJD plane. The starting and end points of the TSO or PFTJ were estimated on axial images. Second, the two orthopedic surgeons evaluated the preoperative MRI images obtained using axial and coronal fat-saturated proton density (PD)-weighted turbo spin-echo sequences and sagittal T2-weighted turbo spin-echo sequences. Third, the corresponding CT and MRI data sets were reviewed by the two orthopedic surgeons. To evaluate the risk of injury to neurovascular structures, the osteotomy or detachment plane identified on the CT scans was embedded in the MRI images on axial and coronal views. For an exact side-by-side comparison, distances from the joint line were matched in both images.

### 2.5. Statistical Analyses

All variables are presented as the mean ± standard deviation. The Mann–Whitney U-test was used to compare the distances from the TSO site or PTFJ to the peroneal nerve and popliteal artery. The protective effect of the popliteus muscle was expressed as a percentage, and the significance of differences between the groups was evaluated using the chi-square test. All statistical analyses were performed using SPSS^®^ (IBM^®^ Corp, Armonk, NY, USA, version 24), and significance was set at *p* < 0.05.

## 3. Results

The TSO procedure was technically straightforward and did not result in incomplete separation. Separation of the osteotomy site could be confirmed fluoroscopically by levering the tibial bone fragment and fibula head laterally using an osteotome. Complete closure of the tibial osteotomy gap was achieved in all 13 cases, avoiding incomplete closure that may occur when the fibula is untethered incompletely. The mean HKA axis and MPTA were changed from 11.0 to 2.0 and from 80.7 to 89.5 after operation.

The distance from the TSO or PTFJD plane to the peroneal nerve on the axial images was significantly longer in the TSO group than the PTFJD group (*p* = 0.001; Table 1). The distance from the TSO or PTFJD plane to the popliteal artery was also significantly longer in the TSO group than the PTFJD group (*p* < 0.001). The intra- and inter-observer reliability for all radiographic measurements was considered acceptable, ranging from 0.81 to 0.99 and from 0.81 to 0.96, respectively.

In all patients in the TSO group, the osteotomy plane was covered by the popliteus muscle in the coronal and axial images. However, in the PTFJD group, the detachment plane was not covered by the popliteus muscle in the coronal or axial plane in all cases (Figure 4D).

## 4. Discussion

The most important finding of this study is that our novel TSO technique is technically straightforward while enabling effective fibular untethering, and it can be performed safely, reducing the risk of peroneal nerve and popliteal neurovascular injury.

Despite evident advantages, the decision to perform LCWHTO as an option to correct medial unicompartmental OA with varus alignment should not be made lightly, especially for novice surgeons, given that the procedure requires additional manipulation of the PTFJ or fibula shaft [7]. Due to the inevitably greater risk of injury to the peroneal nerve and popliteal neurovascular structures when performing an LCWHTO procedure to treat medial unicompartmental OA with varus alignment compared with an MOWHTO, many surgeons are hesitant to choose LCWHTO as their surgical option. While many patients can be treated with an MOWHTO, LCWHTO should be considered among the surgical options, especially for patients with a large correction gap, patients at risk of patellofemoral OA, those with an excessive tibial slope, or for patients for which there are concerns about LLD due to unilateral surgery [5].

Various surgical approaches for fibular untethering have been used to perform LCWHTO safely, including fibular shaft osteotomy, PTFJD, and fibular head resection [2,3,8,9]. However, to the best of our knowledge, to date, no technique has been reported to secure the PTFJ articular cartilage and maintain a safe distance to posterolateral neurovascular structures. Previously reported methods for PTFJ in LCHTO have several technical and clinical limitations. Fibular shaft osteotomy is technically demanding because it requires an additional incision to perform osteotomy of the fibular shaft, and there is a risk of nonunion of the osteotomy site [6]; in addition, the risk of peroneal nerve injury, a fatal complication, is not negligible [9,12]. The PTFJD technique limits the correction angle of LCWHTO [6], which reduces the reproducible gap correction and is also associated with a greater risk of TKA conversion than the fibular head resection technique [15].

In terms of surgical straightforwardness, our novel TSO technique has several advantages over PTFJD. In the process of PTFJD, the anterior and posterior PTFLs should be dissociated to prevent incomplete LCWHTO. If dissociation is incomplete, elevation of the fibula head is limited, and closure of the osteotomy gap is also limited by the remnant tethering effect of the PTFL. It is not possible to definitively prove that the PTFJL has been dissociated during the PTFJD procedure: in such circumstances, the surgeon may repetitively apply a dissociating force to the PTFJ with a blunt osteotome, increasing the risk of iatrogenic popliteal neurovascular injury beyond the popliteus muscle or fibula head fracture. In contrast, during the TSO procedure, surgeons can confirm whether the tibial-side osteo-fragment is freely movable or not by using an image intensifier in real time within the operation room, thus simplifying fibula untethering during LCWHTO and improving the reproducibility of the procedure.

Additionally, unlike PTFJD, during the TSO procedure, surgeons can estimate the depth of the osteotomy using haptic sensing and can therefore feel the osteotome advance through the far cortex, allowing them to stop advancing the osteotome. The surgeon is also able to feel the K-wire penetrate the near cortex and cancellous bone and make contact with the far cortex. In contrast, during the PTFJD procedure, surgeons are not able to employ tactile exploration as the osteotome penetrates into deeper layers. These differences in the utility of the tactile sense could provide the beneficial effect of lessening excessive advancement of the surgical device to a deep layer (Table 2).

To simplify the TSO technique, we recommend several technical tips. It is important to identify the border of the PTFJ to prevent unintended injury to the PTFJ articular cartilage and neurovascular bundle. The PTFJ can be identified more easily if blunt dissection of the anterior PTFL is performed; excessive dissection with a sharp scalpel should be avoided because it could cause injury to the PTFL and articular cartilage of the PTFJ. We also recommend inserting the guiding K-wire 5 mm medial to the PTFJ to sufficiently secure the bone fragment and avoid anterior PTFL injury. The creation of an excessively large bony fragment should also be avoided in order to prevent iatrogenic proximal tibia articular fracture. The guiding K-wire should not penetrate the far cortex of the proximal tibia in order to avoid penetrating the popliteal neurovascular structures. We also advise that the oscillating saw should not be advanced farther than the far cortex, and the final detachment of the far cortex should be performed with an osteotome. Finally, complete detachment should be confirmed using an image intensifier.

When compared with the PTFJD procedure, the novel TSO procedure improves safety by moving the osteotomy site farther from the neurovascular structure and allowing the popliteus muscle to provide protective cover. The shortest distances from the TSO or PTFJD plane to the peroneal nerve and popliteal artery were significantly farther in the TSO group. Moving the surgical site farther away from the neurovascular structure reduces the risk of neurovascular injury. In addition, the TSO technique may better protect the recurrent branch of the anterior tibial artery (ATA); the ATA arises from the popliteal artery just below the popliteus muscle and pierces or passes above the interosseous membrane in a posterior-to-anterior direction, giving rise to the anterior tibial recurrent artery, which ascends from the aperture and anastomoses with the genicular arteries [16]. In the TSO procedure, the osteotomy line is positioned farther from the PTFJ and interosseous membrane and should therefore also be farther from the recurrent branch of the ATA; however, we were unable to confirm positioning because the tiny ATA could not be identified in postoperative MRI, and further studies utilizing angio-CT or cadaver studies are required.

Our novel technique also has a protective effect due to the mechanical blocking effect of the popliteus muscle. According to the findings reported by Parker et al., the popliteal neurovascular bundle is thought to be protected by the popliteus muscle and the posterior tibialis muscle [14]. However, our MRI findings with the knee in full extension or at 90° flexion (a position that many surgeons prefer when performing LCWHTO) indicate that the popliteus muscle does not properly cover the posterior PFTJ in the coronal and sagittal planes. The inferior margin of the popliteus muscle is more proximal than the posterior PFTJ and does not provide any protective cover beyond the PTFJ. However, with our novel technique, the osteotomy line lies more medially and parallel to the PTFJ and is fully covered by the popliteus muscle (Figure 4B,D). Moreover, in the axial plane, the popliteal vessel located on the extended line of the PTFJ is positioned more laterally than the TSO line (Figure 4B), which may elevate the risk of injury to the neurovascular bundle during the dissociation of the PTFJ with the osteotome. However, in the TSO technique, the blunt osteotome is blocked by the popliteus muscle, reducing the risk of popliteal neurovascular injury. As such, by opting for the TSO procedure, inexperienced surgeons may avoid many of the risks presented by the LCWHTO procedure while ensuring effective correction of varus malalignment. Further evaluations, including cadaveric studies or randomized controlled trials with larger cohorts, are required to confirm our findings.

This protective effect could transform LCWHTO. Although some previous studies that included cadaveric angiography reported that during knee flexion, the popliteal neurovascular bundle moves posteriorly at the joint line level [17], other studies employing duplex ultrasound in living patients reported that the popliteal artery is closer to the posterior border of the tibia when the knee is in 90° flexion than in full extension, especially at 1 to 2 cm below the joint line, where coronal osteotomy occurs [18,19]. Notably, MRI studies have reported that knee flexion and the effects of gravity do not guarantee repositioning of the popliteal vessels away from potential harm during surgery [20]. Estimating the exact location of the popliteal artery was not feasible, especially at the level of proximal tibial osteotomy, which is about 2 to 3 cm distal to the joint line [17,18,19,20]. In this circumstance, the protective cover provided by the popliteus muscle could be a key pro of our novel technique to improve the safety of the LCWHTO procedure performed by surgeons to treat medial unicompartmental OA.

Our TSO technique does not violate the PTFJ articular cartilage, which is frequently reported in PTFJ detachment, and can reduce the likelihood of iatrogenic fibular head fracture and consequently reduce the risk of peroneal nerve injury. Instead, we separated the PTFJ directly from the tibial side. Fine TSO may be performed using a guiding K-wire and oscillating saw under an image intensifier. By means of this simple TSO technique, surgeons can overcome the risk of PTFJ cartilage injury that may result in lateral knee pain after LCWHTO. Moreover, there is no need for the additional skin incision and soft tissue dissection that are necessary when performing fibular shaft osteotomy procedures, and the TSO technique may reduce the operation time compared to the fibular shaft osteotomy method. These advantages indicate that the TSO procedure is a viable alternative to lateral closure when performing HTO, especially for novice surgeons.

This study had several limitations. First, this study was confined to an Asian population. Thus, these results might not be generalizable to other ethnicities. Second, a radiographic virtual control group was used to compare the anatomical variables of PTFJD with those of our novel technique; however, additional studies using values obtained from real-world PTFJD procedures are required. In addition, the genuine PTFJD plane could not be confirmed on the postoperative CT because it does not leave a confirmable trace in the radiologic test. Together, these limitations may limit the generalizability of our results.

Despite these limitations, our oblique mini-osteotomy of the proximal tibia technique for the PTFJ in LCWHTO can provide a reproducible and safer surgical method, protecting popliteal neurovascular structures and the recurrent branch of ATA. Further validation through additional studies, including cadaveric investigations, is warranted.

## Figures and Tables

**Table 1:** Distances from popliteal artery or peroneal nerve to osteotomy plane.

	TSO Group	PTFJD Group	*p* Value
**Distance from popliteal artery**	8.5 ± 3.4	0.7 ± 1.5	<0.001
**Distance from peroneal nerve**	26.1 ± 2.8	21.7 ± 2.6	0.001

TSO: tibial-side osteotomy; PTFJD: proximal tibiofibular joint detachment.

**Table 2:** Benefits and limitations.

Benefits	Limitations
Reduced risk of iatrogenic peroneal nerve injury because of relative anatomical location	Risk of iatrogenic injury of PTFJ articular cartilage due to incomplete TSO and/or incorrect direction of TSO
Possible protection of underlying popliteus vessel by popliteal muscle	Possible risk of iatrogenic PTFJ injury due to iatrogenic fracture, especially in patients with osteoporosis
Reduced risk of anterior tibial artery recurrent branch injury because osteotomy plane is located farther away	
Fewer morbidities than for PTFJD, including PTFJ articular cartilage injury or iatrogenic fibula head fracture	
Surgeons can use haptic sense to estimate advancement depth of surgical devices	

TSO: tibial-sided osteotomy, PTFJ: proximal tibiofibular joint, PTFJD: proximal tibiofibular joint detachment.

## Data Availability

The data presented in this study are available upon request from the corresponding author.

## Supplementary Materials

The following supporting information can be downloaded at: https://www.mdpi.com/article/10.3390/medicina61010091/s1, Video S1: Lateral closing wedge high tibial osteotomy.
